# Measurement of the Fluorescence Quantum Yield Using a Spectrometer With an Integrating Sphere Detector

**DOI:** 10.6028/jres.113.004

**Published:** 2008-02-01

**Authors:** Adolfas K. Gaigalas, Lili Wang

**Affiliations:** National Institute of Standards and Technology, Gaithersburg, MD 20899

**Keywords:** absolute quantum yield, fluorescence, integrating sphere, spectrophotometer

## Abstract

A method is proposed for measuring the fluorescence quantum yield (QY) using a commercial spectrophotometer with a 150 mm integrating sphere (IS) detector. The IS detector is equipped with an internal cuvette holder so that absorbance measurements can be performed with the cuvette inside the IS. In addition, the spectrophotometer has a cuvette holder outside the IS for performing conventional absorbance measurements. It is shown that the fluorescence quantum yield can be obtained from a combination of absorbance measurements of the buffer and the analyte solution inside and outside the IS detector. Due to the simultaneous detection of incident and fluorescent photons, the absorbance measurements inside the IS need to be adjusted for the wavelength dependence of the photomultiplier detector and the wavelength dependence of the IS magnification factor. An estimate of the fluorescence emission spectrum is needed for proper application of the wavelength-dependent adjustments. Results are presented for fluorescein, quinine sulfate, myoglobin, rhodamine B and erythrosin B. The QY of fluorescein in 0.1 mol/L NaOH was determined as 0.90±0.02 where the uncertainty is equal to the standard deviation of three independent measurements. The method provides a convenient and rapid estimate of the fluorescence quantum yield. Refinements of the measurement model and the characteristics of the IS detector can in principle yield an accurate value of the absolute fluorescence quantum yield.

## 1. Introduction

Integrating spheres (IS) have been used extensively for measuring optical properties of surfaces, films and light sources. The light from a source placed at the center of the sphere is reflected many times by the surface of the IS. By the time the light hits the detector (placed in the wall of the IS) much of the original emission anisotropy has been eliminated so that the detector gives a good measure of the total emitted radiant power. The detailed description of the reflection process inside the IS can be found in the literature [1,2]. The IS technique has been applied to the measurement of absorbance of materials. The benefit of this approach is the lack of sensitivity to scattering and the geometry of the sample [3]. The IS technique was used to measure the absorbance of pure water [4,5]. Recently, the IS technique has been applied to the measurement of quantum yield (QY) from films and solutions. A summary of the traditional methods for measuring QY has been given by Demas [6]. A popular method is the comparison of fluorescence emission spectra from two solutions one of which is a standard. QY measurement with IS of anisotropic films were first discussed by DeMello [7]. It was shown that the results of several measurements with the sample outside and inside the IS could be combined to yield the absolute QY. The procedure was adopted to a commercial fluorometer by Porres [8]. A custom-made fluorometer with an IS has been described by Mei [9]. This work proposes a technique to measure the absolute QY of fluorophores in solution by using a commercial spectrophotometer with an IS accessory. In the following we develop the mathematical model of the technique and then apply it to several fluorophores. With some care, the technique could be used as a convenient method for estimating the absolute QY in the wavelength range of 300 nm to 700 nm.

## 2. Methodology

**Fig. 1:**
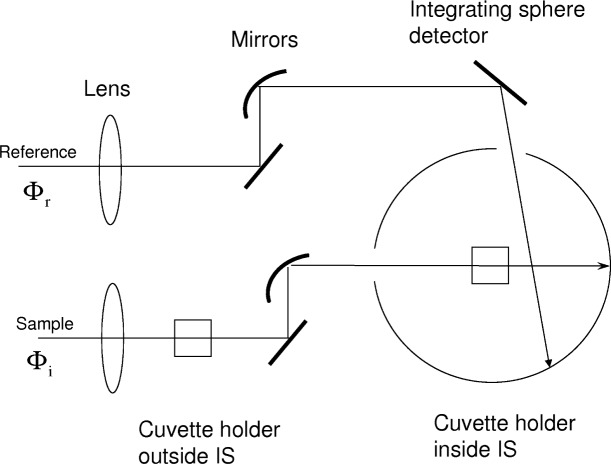
A schematic diagram of the geometric layout of the reference and sample beam paths in the Lambda 850 spectrometer with the integrating sphere (IS) detector. The two squares represent the cuvette holders placed in the path of the sample beam. One holder is outside the IS, and the other holder is inside the IS located at the center of the sphere. Without cuvettes in the holders, the responses of the IS detector to the reference and sample beams are almost identical.

The proposed method for measuring the fluorescence quantum yield utilizes a commercial spectrophotometer with an integrating sphere (IS) detector. Two measurements of absorbance have to be carried out on the same cuvette. One measurement is performed with the cuvette placed outside the IS, and the second measurement with the cuvette inside the IS. For clarity, the measurement will be described with a specific instrument—the Perkin Elmer Lambda 850^1^. Figure 1 shows the paths of the sample and reference beams used in the measurement of absorbance. The two paths are very similar; both end on the wall of the IS detector. The path of the sample beam contains two mechanical holders for a cuvette. One cuvette holder is located in front of the IS detector while the second cuvette holder is located inside the IS detector. The lens and the mirrors shape the sample beam so that it passes unobstructed through the cuvette holders.

**Fig. 2:**
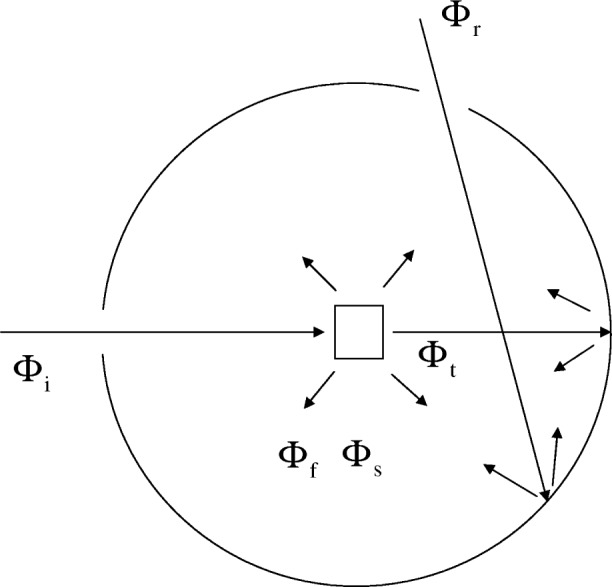
Top view of the IS detector. The arrows show the various light fluxes present in the IS when a cuvette containing a fluorescent solution is placed in the holder inside the IS. Φ_i_ and Φ_r_ are the fluxes that enter the IS through the sample port and reference port respectively. Note that the reference flux does not pass through the cuvette at the center of the IS and hits the inside surface of the IS. The flux, Φ_i_, passes through the cuvette and its contents. The passage results in three new fluxes: Φ_t_ the flux transmitted through the cuvette, Φ_f_ the fluorescence flux, and Φ_s_ the flux due to scattering from the cuvette walls. There are baffles (not shown) placed in the IS to prevent the detector from seeing the first reflection of the transmitted and reference beams as well as the direct observation of the fluorescence and scattered fluxes. The detector is sensitive to average fluxes resulting after many reflections from the surface of the IS.

An incident flux, Φ_i_ (W-Watts), enters the IS through a sample aperture of area *A*_i_ (m^2^), passes through a cuvette, and hits the wall of the IS. A reference flux, Φ_r_ (W), enters the IS through a reference aperture of area *A*_r_ (m^2^) and hits the IS wall without passing through the cuvette. Figure 2 illustrates the geometrical arrangement of the various fluxes inside the IS. First consider the reference flux. The reference beam will enter the IS, hit the IS wall and undergo the first reflection. A baffle inside the IS prevents the detector from seeing the first reflection of the incident reference beam. The reference light beam undergoes many more reflections resulting in an average flux, $\Phi_{\mathrm{ref}}^{s}$, incident on the IS wall. The average flux will be written as

$$\Phi_{\mathrm{ref}}^{s}=M_{\mathrm{ref}}\Phi_{r},$$ (1)

where the symbol *M*_ref_ represents the magnification of the incident reference flux due to the many reflections on the IS wall. Neglecting the presence of the cuvette holder and the baffle, the ideal IS behavior leads to the following expression for the magnification *M*_ref_ = *ρ*(1 − *f*)/(1 − *ρ*(1 − *f*)) [10]. The constant *f* is defined as the ratio of areas given by *f* = (*A*_i_ + *A*_r_ + *A*_d_)/*A*_S_ where *A*_d_ and *A*_S_ are the areas of the detector aperture and total sphere surface respectively. The reflectance of the material lining the surface of the IS is given by *ρ*. The detector is mounted flush with the surface of the IS. Therefore the detector response will be proportional to the average flux incident per unit surface area of the IS multiplied by the detector area. Explicitly, the detector signal, *D*_ref_, will be given by Eq. (2)

$$D_{\mathrm{ref}}=R(\lambda )\Phi_{r}M_{\mathrm{ref}}\frac{A_{d}}{A_{s}}\equiv E(\lambda )M_{\mathrm{ref}}\Phi_{r},$$ (2)

where *R*(*λ*) is the radiant sensitivity of the photomultiplier cathode. The ratio of the detector area to the area of the entire IS is a constant which can be combined with the radiant sensitivity to give overall detection efficiency *E*(*λ*). It will be assumed that the dependence of the detection efficiency on wavelength comes from the radiant sensitivity of the photomultiplier cathode. An excellent description of various biases inherent in IS measurements has been presented by Hanssen and Snail [11].

Consider the detector response for the case of the flux, Φ_i_, entering the IS through the sample port and hitting the cuvette filled with a fluorescing solution. As shown in Fig. 2, there is a flux associated with the incident flux which is transmitted through the cuvette. This flux will be called Φ_t_ (W). Another flux will arise due to scattering from the cuvette walls. The scattering flux will be called Φ_s_ (W). Finally there will be a flux associated with the radiative decay of excited molecules. The fluorescence flux will be emitted over an extended range of wavelengths and will be called Φ_f_ (W/nm). The scattered and fluorescence fluxes are emitted from the cuvette in all directions. The cuvette holder inside the IS has a bottom coated with the same material as the surface of the IS. This bottom serves as a “baffle” preventing the detector from seeing the direct fluorescence and scattered fluxes. Therefore the fluorescence and scattered fluxes arriving at the detector will be the average fluxes obtained after many reflections. However, the fluorescence flux is spread over the wavelength region associated with the fluorescence emission spectrum and therefore it has different detection efficiency from that of the scattered flux. All of these considerations lead to the following expression for the detected signal, *D*_sample_, when the flux Φ_i_ enters the IS through the sample port.

$$D_{\mathrm{sample}}=E(\lambda )M_{\mathrm{inc}}\Phi_{t}+\int_{\lambda_{f}}E(\lambda_{f})M_{\mathrm{cuv}}\Phi_{f}(\lambda_{f})d\lambda_{f}+E(\lambda )M_{\mathrm{cuv}}\Phi_{s}.$$ (3)

*M*_inc_ is the magnification factor for flux transmitted through the cuvette. The transmitted flux hits the same spot on the surface of the IS as the incident flux, and therefore it is likely that it evolves identically to the incident flux in the absence of a cuvette. *M*_cur_ is the mul tiplication factor for flux originating in the cuvette. It is assumed that the fluorescence and scattered fluxes evolve in similar fashion. Equation (3) models the response of the IS to the three fluxes which ultimately arise from the perturbation of the incident flux by the cuvette. The relation between the incident flux, Φ_i_, and the fluxes exiting the cuvette will be determined by the cuvette and its contents as discussed below. Finally, if there is no cuvette in the sample holder, then *D*_sample_ and *D*_ref_ should in principle be equal because the incident flux is not modified. The autozero function of the Lambda 850 corrects for small differences in IS response between the reference beam and the unobstructed sample beam. Equations (2) and (3) suggest that to a very good approximation balancing the detector signals is equivalent to balancing the two average fluxes, *M*_inc_Φ_i_ = *M*_ref_Φ_r_. Subsequent to the autozero, and the insertion of a cuvette into the sample holder, the Lambda 850 measures the average fluxes incident on the IS surface and reports the absorbance, *A*, calculated from the relation *A* = −log(*D*_sample_/*D*_ref_). This procedure is carried out irrespective of whether the cuvette is placed inside or outside the IS. In what follows we will use Eqs. (2) and (3), and the measured values of *A* to extract the fluorescence quantum yield. We start with the discussion of traditional absorbance measurement with the cuvette mounted in a holder outside the IS.

### 2.1 Cuvette Outside IS

**Fig. 3:**
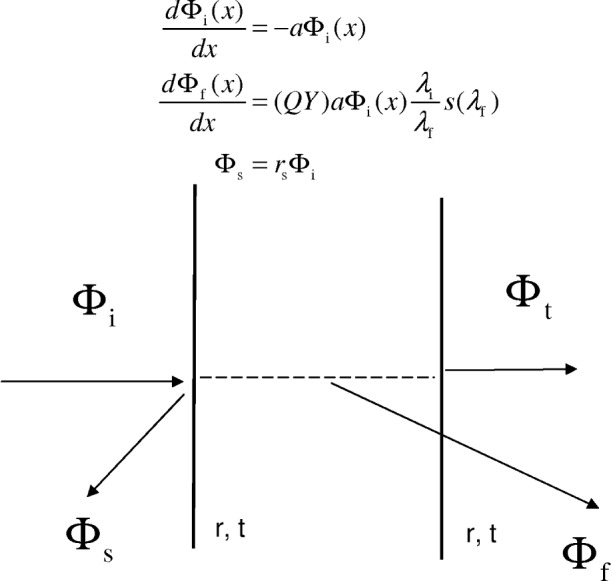
A model of the interaction of the incident flux with the contents of the cuvette. The incident beam is attenuated in the cuvette by molecular absorption of light. Whatever flux remains after the passage through the cuvette is called the transmitted flux. The fluorescence flux is represented as a fraction of the absorbed flux where the fraction is called the quantum yield (QY). The scattering flux is represented as a constant fraction of the incident flux.

Figure 3 shows a model of the expected response when a beam of light passes through a cuvette filled with a fluorescing solution. The two vertical lines in Fig. 3 represent the walls of the cuvette and the horizontal arrows represent the incident and transmitted flux. The molecular absorption coefficient, *a* = *σN*, is expressed as a product of the absorption cross section, *σ*, in units of cm^2^ and the molecular concentration, *N*, in units of cm^–3^. The fluorescence flux is modeled as originating from the absorbed flux with a parameter called the quantum yield (QY) which gives the fraction of absorbed photons that appear as fluorescence photons. For the case of a cuvette outside the IS detector, we assume that the amount of fluorescence flux that reaches the detector is negligible. This assumption is plausible since the fluorescence is emitted in all directions and only a negligible part will be accepted by the detector aperture. Let *r* and *t* be the reflection and transmission coefficients of the two cuvette walls. The effect of the first cuvette wall is represented by a change in the incident flux to *t*Φ_i_ = 10^log^*^t^*Φ_i_. The differential equation can be solved to find the flux after the beam has traversed the fluid inside the cuvette. The result is *t*Φ_i_*e*^−al^ = *t*Φ_i_10^−0.434al^ where *l* is the path length which will be set to 1 cm. The second cuvette wall introduces another transmission coefficient. Finally the transmitted flux can be written in terms of the incident flux and the properties of the cuvette to give the ratio of expected signals shown in Eq. (4)

$$\begin{array}{l} \frac{D_{\mathrm{sample}}}{D_{\mathrm{ref}}}=\frac{M_{\mathrm{inc}}\Phi_{t}}{M_{\mathrm{ref}}\Phi_{\mathrm{ref}}}=10^{\log t-0.434a+\log t}\\ \frac{D_{\mathrm{sample}}}{D_{\mathrm{ref}}}\equiv 10^{-A^{\mathrm{ou}t}}. \end{array}$$ (4)

The three terms in Eq. (4) represent the sequential reduction of the incident flux due to the transmission through the first cuvette wall, the transmission through the fluid, and the transmission through the second wall. The exponential notation permits a convenient addition of the effects in the absorbance *A*. The value of *A* presented by the instrument can be compared directly to the exponent given by the model in Eq. (4). A measurement of the absorbance with buffer in the cuvette would give just the “absorbance” due to the finite transmittance at the cuvette walls, which can be subtracted from the measured absorbance of the analyte. In principle it is necessary to consider multiple reflections in the cuvette which would increase the transmitted flux. At this point, the effect of the multiple reflections will be neglected since for typical values of *t* and *a*, the contribution from the multiple reflections is less than 0.0005 absorbance units.

### 2.2 Cuvette Inside the IS

The measurement of absorbance with the cuvette inside the IS is a very different process since the measured flux includes the transmitted flux as well as fluorescence and scattered fluxes. The fluxes are shown in Fig. 2 which gives a scheme of the IS layout. Note that each of the fluxes originating in the cuvette (transmitted, reflected, and fluorescence) goes directly to the IS surface where it is further reflected.

It will be assumed that the incident flux reflected from the first and second cuvette walls exits the IS through the entrance aperture. The design of the IS and the shape of the entrance beam are optimized to insure this event. The sample beam entering the IS detector is focused on the back surface of the IS. This means that the beams reflected from the cuvette walls (located in the center of the IS) are focused on the entrance aperture of the IS detector. Therefore the cross sectional area of the reflected beams at the entrance aperture of the IS will be smaller than the cross section of the incident beam, and therefore all of the reflected beams should exit the IS detector.

The fluorescence flux from a slab of thickness d*x* of the material in the cuvette can be modeled as $d\Phi_{f}=(\mathrm{QY})a\Phi_{i}(x)\frac{\lambda_{i}}{\lambda_{f}}s(\lambda_{f})dx$ where (QY) is the fluorescence quantum yield, and a is the molecular absorption coefficient. The resulting fluorescence flux can be expressed as follows. Let Φ_i_ (W) represent the energy flux incident on a cuvette. Then the transmitted flux is given by 10^−0.434^*^a^*Φ_i_*t*. The energy absorbed in the cuvette is given by (1 − 10^−0.434^*^a^*)Φ_i_*t*. In practice the incident light is reasonably monochromatic. Therefore it is possible to calculate the total number of incident photons absorbed by the material in the cuvette as (1 − 10^−0.434^*^a^*)Φ_i_*t*/*hf*_i_ where *hf*_i_ is the energy of a single incident photon. The quantum yield, QY, gives the fraction of photons emitted for a given number of absorbed photons. Therefore the number of emitted photons is given by QY(1 − 10^−0.434^*^a^*)Φ_i_*t*/*hf*_i_. The emitted photons are usually distributed over a range of wavelengths. The quantity *s*(*λ*f) (nm^−1^) gives the fraction per unit wavelength of the total emitted photons with a wavelength *λ*f. Therefore the number of photons emitted per unit wavelength is given by QY(1 − 10^−0.434^*^a^*)*t*Φ_i_*s*(*λ*_f_)/*hf*_i_. The energy emitted per unit wavelength is obtained by multiplying the number of photons by the energy of a single emitted photon. The final result for the fluorescence flux per unit wavelength (W/nm) is given by

$$\Phi_{f}(\lambda_{f})=\mathrm{QY}(1-10^{-0.434a})t\Phi_{i}s(\lambda_{f})\frac{\lambda_{i}}{\lambda_{f}},$$ (5)

where the ratio of photon frequencies has been replaced by a ratio of the wavelengths. The path length, *l*, was set to 1 cm. The increase in the fluorescence flux due to multiple reflections inside the cuvette can be modeled by a beam propagating perpendicularly to the cuvette walls. Multiple reflections result in an increase of about 4 % in the fluorescence flux. This effect will not be included in this initial presentation. The fluorescence flux may be reduced by self absorption since most fluorophores exhibit a slight overlap between absorption and emission bands. This effect will also be neglected. The scattered flux from the cuvette walls will be modeled as a constant fraction of the incident flux, Φ_S_ = *r*_S_Φ_i_. The ratio of the detector responses when the beam enters through the sample port and when it enters through the reference port can be modeled by inserting the values of the specific fluxes into Eq. (3) and dividing by Eq. (2). The result is given explicitly in Eq. (6)

$$\frac{D_{\mathrm{sample}}}{D_{\mathrm{ref}}}=10^{2\log(t)-0.434a}+\mathrm{QY}(1-10^{-0.434a})\frac{t\cdot \lambda }{E(\lambda )M_{\mathrm{inc}}}\int_{\lambda_{f}}E(\lambda_{f})M_{\mathrm{cuv}}\frac{s(\lambda_{f})}{\lambda_{f}}d\lambda_{f}+\frac{M_{\mathrm{cuv}}}{M_{\mathrm{inc}}}r_{s}\frac{D_{\mathrm{sample}}}{D_{\mathrm{ref}}}\equiv 10^{-A^{\mathrm{in}}}.$$ (6)

Together with Eq. (4), Eq. (6) suggests an explicit relation between the QY and the measured quantities.

$$\mathrm{QY}=\frac{\left(10^{-A_{\mathrm{sol}}^{\mathrm{in}}}-10^{-A_{\mathrm{sol}}^{\mathrm{out}}}-(M_{\mathrm{cuv}}(\lambda )/M_{\mathrm{inc}}(\lambda ))r_{s}\right)}{(1-10^{-0.434a})t}\frac{E(\lambda )\cdot M_{\mathrm{inc}}(\lambda )}{\lambda \cdot \langle EM\rangle }.$$ (7)

Equation (7) serves as the fundamental relation between the measured absorbencies and the fluorescence QY. Note that the dependence on the wavelength of all quantities has been made explicit. The quantities on the right hand side of Eq. (7) can in principle be measured.

$$\begin{array}{c} 0.434\cdot a=A_{\mathrm{sol}}^{\mathrm{out}}-A_{\mathrm{buf}}^{\mathrm{out}}\\ t=10^{-A_{\mathrm{buf}}^{\mathrm{out}}/2}\\ \left(M_{\mathrm{cuv}}(\lambda )/M_{\mathrm{inc}}(\lambda )\right)r_{s}=10^{-A_{\mathrm{buf}}^{\mathrm{in}}}-10^{-A_{\mathrm{buf}}^{\mathrm{out}}}\\ \langle EM\rangle =\int_{\lambda_{f}}\frac{E\left(\lambda_{f}\right)}{\lambda_{f}}M_{\mathrm{cuv}}\left(\lambda_{f}\right)s\left(\lambda_{f}\right)d\lambda_{f}. \end{array}$$ (7a)

The notation in Eq. (7) uses the subscript to specify the solution that is measured, and the superscript to indicate whether the measurement was carried out inside the IS (in) or outside the IS (out).

It is useful to note that Eq. (7) is equivalent to the usual definition of the fluorescence quantum yield. For simplicity, we assume an ideal case where there is complete transmission through the walls of the cuvette, no scattered flux (*t* = 1, *r*_s_ = 0, *δM* = 1), and constant detection efficiencies over all wavelengths. In this ideal case, Eq. (7) reduces to Eq. (8)

$$\mathrm{QY}=\frac{10^{-A^{\mathrm{in}}}-10^{-A^{\mathrm{out}}}}{1-10^{-A^{\mathrm{out}}}}\frac{\langle \lambda_{f}\rangle }{\lambda }.$$ (8)

The expression $\left(1-10^{-A^{\mathrm{out}}}\right)\lambda$ gives the fraction of incident photons which were absorbed by the analyte. The expression $\left(10^{-A^{\mathrm{in}}}-10^{-A^{\mathrm{out}}}\right)\langle \lambda_{f}\rangle$ gives the fraction of the absorbed photons which were converted into fluorescence photons. Thus Eq. (8) follows the standard definition of QY as the ratio of emitted photons to absorbed photons. Note that the emitted photons could originate from both the singlet and triplet excited states of the molecule. Therefore the measured QY describes the total photon emission.

In the following, we perform some measurements and demonstrate that the analysis of the data with Eq. (7) and Eq. (7a) does lead to a good estimate of the fluorescence QY. In the process, we explain how to estimate the overall detection efficiency factor defined as *E*(*λ*)·*M*_inc_(*λ*)/(〈*EM*〉 ·*λ*).

## 3. Results

We describe the application of Eq. (7) and Eq. (7a) to the absorption spectra of fluorescein, quinine sulfate, rhodamine B, erythrosin B, and myoglobin. These fluorophores cover a wide range of quantum yields and provide a good test of the proposed measurement technique for fluorescence QY. The results for fluorescein are presented in detail, and some results for quinine sulfate are shown. The data for the other fluorophores are not shown; the results for all fluorophores are summarized in Table 1.

### 3.1 Fluorescein

**Fig. 4:**
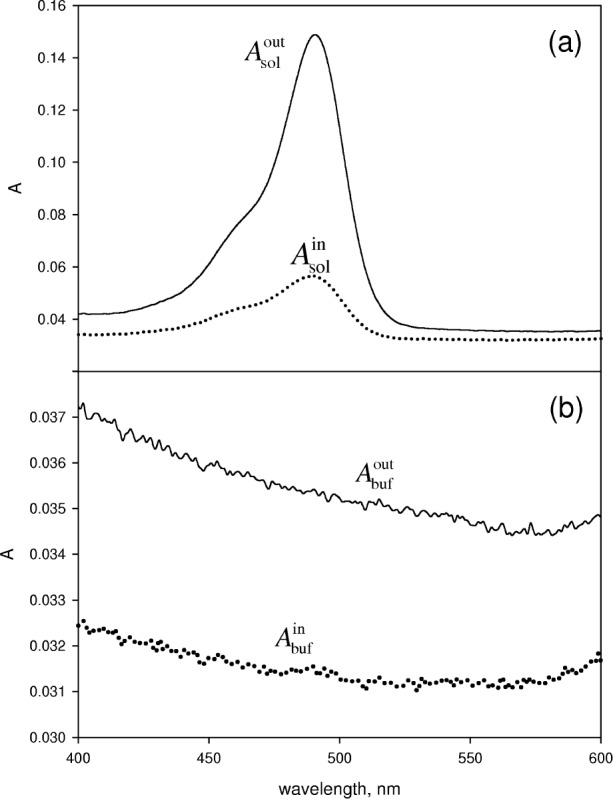
Absorbance spectra of fluorescein. (a) The solid curve shows the absorbance resulting from a fluorescein solution in a cuvette placed outside the IS detector. The dotted curve shows the absorbance when the same cuvette is transferred to the holder inside the IS detector. The reduction in apparent absorbance is a direct consequence of fluorescence. (b) The solid curve shows the absorbance measured in a cuvette filled with buffer and placed outside the IS. The dotted curve shows the absorbance when the cuvette is transferred to the holder inside the IS detector. The reduction in the apparent absorbance is a result of scattering from the cuvette. The design of the IS detector insures that the flux reflected from the cuvette walls exits the IS through the sample port.

**Fig. 5:**
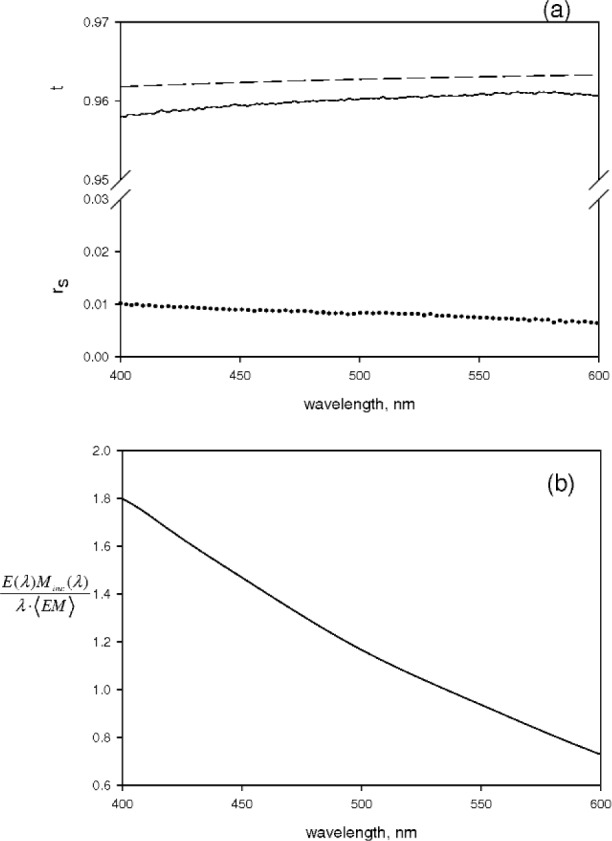
(a) The solid curve and the dotted curve show the transmission coefficient and the scattered flux respectively as obtained from the data in Fig. 4 and Eq. (7a). The dashed curve shows the expected transmission coefficient calculated using Eq. (9). The smaller observed transmission is most likely due to scattering from the cuvette walls. (b) The solid curve shows the ratio of detection efficiencies of the incident flux and the average fluorescence flux. The values were obtained from Eq. (10) as described in the text. The detection efficiency correction is a strong function of the wavelength.

The solid line in Fig. 4a shows the absorbance measured outside the IS detector for a 1.2 μmol/L solution of fluorescein in 0.1 mol/L NaOH solution. The dotted line shows the absorbance measured for the same solution inside the IS detector. The two spectra are averages of three independent measurements. The type A measurement uncertainties are of the order of the thickness of the line in Fig. 4a. The solid line and the dotted line in Fig. 4b shows the absorbance of a 0.1 mol/L NaOH solution measured outside and inside the IS detector respectively. The displayed spectra are averages of three independent measurements. The four spectra in Fig. 4 are the primary measurements which are used in Eq. (7) and Eq. (7a) to obtain the fluorescence quantum yield. The transmission coefficient obtained from Eq. (7a) is shown by the solid line in Fig. 5a. The dashed line in Fig. 5a shows the expected values of *t*. The transmission coefficient is a product of two transmission coefficients associated with the air-fused silica and fused silica-water interfaces. These two transmission coefficients are evaluated using the well known expression given in Eq. (9) with the result shown by the dashed line in Fig. 5a

$$\begin{array}{r} t_{\text{air-fused}\;\mathrm{silica}}=\frac{4\mu_{1}}{{\left(1+\mu_{1}\right)}^{2}}\;,\;\mu_{1}=\frac{n_{\mathrm{fused}\;\mathrm{silica}}}{n_{\mathrm{air}}}\\ t_{\mathrm{fused}\;\mathrm{silica}-\mathrm{water}}=\frac{4\mu_{2}}{{\left(1+\mu_{2}\right)}^{2}}\;,\mu_{1}=\frac{n_{\mathrm{water}}}{n_{\mathrm{fused}\;\mathrm{silica}}}\\ t=t_{\mathrm{air}-\mathrm{fused}\;\mathrm{silica}}\cdot t_{\mathrm{fused}\;\mathrm{silica}-\mathrm{water}}.\; \end{array}$$ (9)

The indexes of refraction are functions of the wavelength given by *n*_fused silica_(*λ*) = 1.561 − 3.969·10^−4^*λ* + 5.183·10^−7^*λ*^2^ − 2.384·10^−10^*λ*^3^ [12] and *n*_water_(*λ*) = 1.3231 + 3300·*λ*^−2^ − 3.2·10^7^*λ*^−4^ [13]. As seen in Fig. 5a, the correspondence between the measured and calculated values of *t* is reasonable. The calculated value of *t* shown in Fig. 5a corresponds to the optimal case of a perfectly smooth and clean surface. The measured values of *t* are slightly smaller indicating some additional loss, most likely due to scattering from the interfaces. The dotted line in Fig. 5a shows the contribution from “scattering” obtained using Eq. (7a). Some of this “scattering” could be due to imperfections in the cuvette surface. It should be mentioned that the “scattering” contribution increased dramatically if the cuvette was not perfectly seated in the cuvette holder inside the IS. (This is most likely due to some of the reflected beam hitting the surface of the IS). Monitoring the “scattering” contribution at a wavelength where there is no molecular absorption yields a good indicator of proper cuvette seating in the IS holder.

The relative cathode radiant sensitivity curve, as given in the Hamamatsu data sheet for the R928 photomultiplier tube (PMT), was read at selected values of the wavelength and these values were used to interpolate the cathode radiant sensitivity at arbitrary values of the wavelength. The resulting relative cathode radiant sensitivity is given in Table 2. These values were used for the analysis of all measurements. The emission spectrum of fluorescein was measured using an SLM 8000 fluorometer. The average of the product of magnification and the cathode radiant sensitivity for fluorescence was calculated using Eq. (10)

$$\langle EM\rangle =\frac{\int_{400}^{700}spect\left(\lambda_{f}\right)\frac{E\left(\lambda_{f}\right)}{\lambda_{f}}M_{\mathrm{cuv}}\left(\lambda_{f}\right)d\lambda_{f}}{\int_{400}^{700}spect\left(\lambda_{f}\right)d\lambda_{f}}.$$ (10)

**Fig. 6:**
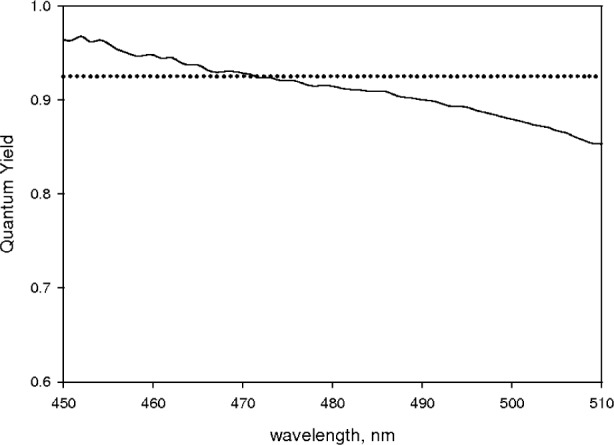
The solid curve shows the values of QY of fluorescein as determined from Eq. (7) and Eq. (7a). As discussed in the text, the ratio of the magnification factors of the incident flux and the average fluorescence flux was set to 0.94 which is independent of wavelength. The resulting value of QY at 490 nm is 0.90 which is lower than the expected value shown by the dotted curve.

The symbol *spect*(*λ*_f_) is a continuous emission spectrum obtained by interpolation of the measured uncorrected emission spectrum. The emission spectrum was measured using a photon counting detector so that the value of *spect*(*λ*_f_) at any wavelength is proportional to the number of emitted photons at that wavelength. The spectral function *s*(*λ*_f_) was estimated from the relation *s*(*λ*_f_) = *spect*(*λ*_f_)/∫*spect*(*λ*_f_)d*λ*_f_. *E*(*λ*) represents the interpolated values of the relative cathode radiant sensitivity data given in Table 2 (obtained from the PMT data sheet). The resulting detection efficiency factor, *E*(*λ*)·*M*_inc_(*λ*)/(*λ* ·〈*ΕΜ*〉), is shown by the solid line in Fig. 5b (it was assumed that the magnification ratio, *M*_cur_/*M*_inc_, was a constant equal to 0.94 as discussed below). Combining all of the measurements, the final values of the quantum yield obtained using Eq. (7) and Eq. (7a) are shown by the solid line in Fig. 6. The dotted line shows the expected result [14].

The correspondence of the measured and expected values of quantum yield in Fig. 6 is reasonably good. However the wavelength dependence of the measured values of quantum yield suggests that the calculated detection efficiency factor does not represent correctly all of the wavelength dependence. For the previous analysis to be valid, the magnification ratio, *M*_cuv_/*M*_inc_, has to be relatively constant over the spectral region of interest (e.g. 480 nm to 600 nm). To obtain an estimate of the magnification ratio, a source of known flux has to be placed inside the cuvette. A possible source is a slab of Spectralon placed diagonally in a cuvette filled with water. The measured absorbance for this cuvette inside the IS can be calculated using Eq. (11)

$$10^{-A_{s\mathrm{pectralon}}^{\mathrm{in}}}=\delta M\cdot t\cdot \rho +\delta M\cdot r_{s}.$$ (11)

**Fig. 7:**
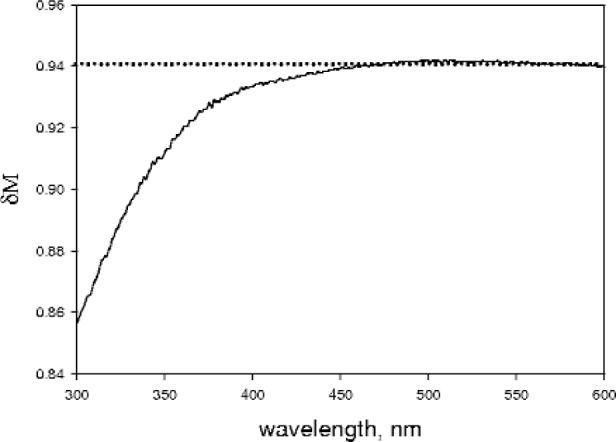
The solid line shows the estimate of the ratio of magnification factor of the flux originating in the cuvette to the magnification factor of the flux hitting the IS surface. The curve was obtained using Eq. (11) and measurements of a Spectralon slab placed diagonally inside a cuvette filled with water. The ratio is a constant down to a wavelength of 450 nm. For lower wavelengths, the ratio decreases. The dotted curve gives the magnification factor for a constant value of 0.94.

The symbol *δM* stands for the ratio of magnification for fluxes originating in the cuvette to the magnification for the flux hitting the IS surface, *M*_cur_(*λ*)/*M*_inc_(*λ*). The transmission and scattering contributions can be determined by measuring the absorbance of a cuvette filled with water outside and inside the IS [see Eq. (7a)]. This procedure leads to an estimate of the magnification ratio shown in Fig. 7. The solid curve in Fig. 7 shows the average of two measurements carried out for the two possible orientations of the Spectralon slab in the cuvette. The dotted curve in Fig. 7 is a straight line drawn to guide the eye in estimating deviation from a constant value. The magnification ratio is about 0.94 for wavelengths greater than 450 nm. For shorter wavelengths the magnification ratio decreases to 0.86 at 300 nm. Since most of the fluorescence emission from fluorescein is above 450 nm, the magnification ratio averaged over the emitted fluorescence spectrum should be close to 0.94, the value used in the fluorescein data analysis.

The magnification of the flux originating in the cuvette is related to magnification of the incident beam by *M*_cur_ = *δM*·*M*_inc_. Therefore the measurement of *δM* may not take into account all of the wavelength dependence. It is also necessary to estimate the wavelength dependence of *M*_inc_. The magnification is known to be a sensitive function of the reflectance of the material lining the surface of the IS. Table 3 shows rough estimates of the IS reflectance in the near UV obtained from the graphs in manuals provided by the manufacturer. The reflectance values are “generic” and may be slightly different from the values associated with the particular instrument. The values of magnification shown in Table 3 were obtained using the function *M*_inc_ = *ρ*(1 − *f*)/(1 − *ρ*(1 − *f*)) which is valid for the behavior of an ideal IS. Clearly the magnification is a sensitive function of the reflectance. The change in reflectance from 500 nm to 300 nm is only 3 % but the resulting magnification varies by as much as 57 %. Most likely the additional wavelength dependence of *M*_inc_ could introduce a modification of the QY results of fluorescein shown in Fig. 6. A more accurate estimate of the dependence of the magnification on wavelength is needed for an improved analysis.

### 3.2 Rhodamine B, Erythrosin B, and Myoglobin

**Fig. 8:**
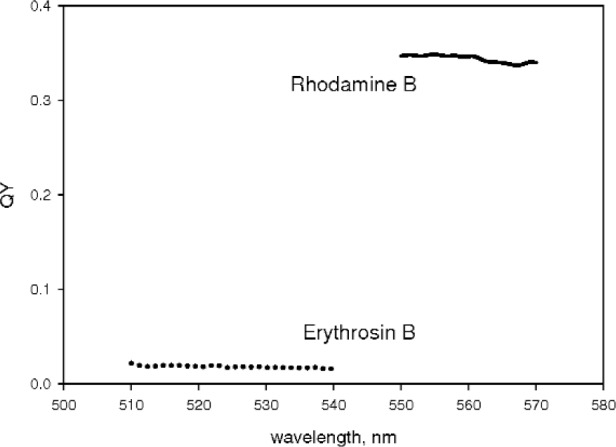
The solid curve shows the measured quantum yield of rhodamine B in the vicinity of the maximum absorption. The procedure used was identical to that used to generate the quantum yield of fluorescein shown in Fig. 6. The dotted curve shows the quantum yield of erythrosin B in the vicinity of maximum absorption. Both values of QY agree with published values.

Figure 8 shows the QY obtained for rhodamine B and erythrosin B using the identical procedure described for fluorescein. The quantum yield of myoglobin was consistent with 0.0. In all three cases, the shape of the absorbance spectra measured inside and outside the IS were very similar. The similarity is expected for erythrosin B and myoglobin since they have small and vanishing fluorescence emission respectively. The emission of rhodamine B falls on the part of the detection efficiency curve where it decreases linearly with wavelength. This is expected to cause only a small distortion of the absorbance spectrum measured inside the IS (similar to fluorescein). The application of the same data analysis procedure as discussed for fluorescein gave a QY of 0.34 ± 0.02 for rhodamine B at 560 nm. The result for erythrosine B was QY = 0.018 ± 0.02 at 530 nm, and for myoglobin QY = −0.01. In the case of myoglobin, the absorbances measured outside and inside the IS are so close in value that the final result for QY is very sensitive to the values of the measured transmission and scattering contributions. Both of the latter quantities are somewhat noisy and susceptible to other confounding factors such as reproducibility of the cuvette position in the holder. The results for myoglobin suggest that the reference beam is not modified appreciably when the cuvette with the myoglobin solution is placed inside the IS. In principle the reference beam could be attenuated due to absorption by myoglobin. However this effect appears to be minimal.

The measurement of the QY of fluorescein, rhodamine B, and erythrosin B give values of 0.90, 0.34, and 0.018 that are close to the accepted values of 0.925, 0.31, and 0.02 respectively. This suggests that the proposed technique works for wavelengths above 450 nm. However the analysis of the data at lower wavelengths is complicated by the strong dependence of magnification on wavelength.

### 3.3 Quinine Sulfate

**Fig. 9:**
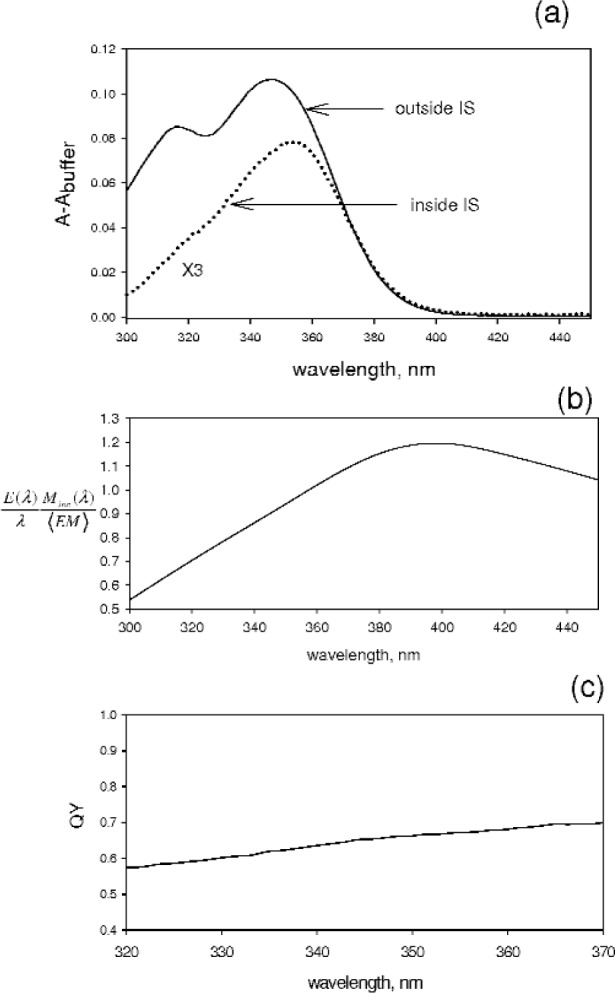
(a) The solid curve shows the absorbance resulting from a quinine sulfate solution in a cuvette placed outside the IS detector. The dotted curve shows the absorbance (X3) when the same cuvette is transferred to the holder inside the IS detector. The reduction in apparent absorbance is a direct consequence of fluorescence while the change in the spectral shape is most likely due to wavelength dependence of the detection efficiency and the magnification ratio. (b) Calculation of the detection efficiency factor in Eq. (7) and Eq. (7a) assuming ideal relationship between the reflectance and the magnification factor. The wavelength dependence of the reflectance was taken from generic data presented in the Labsphere, Inc. manual. The large wavelength dependence is largely due to the magnification factor. (c) Estimated QY of quinine sulfate. The value is higher than the accepted value. However most of the wavelength dependence in the spectra of part (a) has been accounted for.

Figure 9a shows the absorbance measured for a 1 μmol/L solution of quinine sulfate in 0.1 mol/L H_2_SO_4_. The solid curve shows the absorbance measured outside the IS, and the dotted curve shows the absorbance (multiplied by 3) measured inside the IS. In both cases the contribution from the buffer has been subtracted. There is a substantial difference in the shape of the spectrum between the two measurements. This difference can be rationalized by the presence of fluorescence emission in the case of measurements inside the IS. Due to the strong wavelength dependence of the magnification, the fluorescence emission has significantly higher detection efficiency factor. The values of the transmission coefficient and the scattering contribution are very similar to those shown in Fig. 5a although the transmission factor is 0.958 at 300 nm. The most likely cause of the large wavelength dependence of the difference in the shape of the absorption spectrum is the change in the magnification factor resulting from the decrease in reflectivity at lower wavelengths. An estimate of the detection factor for quinine sulfate was made using the data in Table 2 and Table 3. The solid curve in Fig. 9b shows the final value of the detection factor. The calculated detection factor in conjunction with the absorbance measurements gave a QY (shown in Fig. 9c) 0.65 ± 0.03 at 350 nm where the uncertainty was obtained from repetitive measurements. This value of QY is larger than the accepted value of 0.58 [15] indicating the presence of substantial systematic error. It was found that the value of QY was very sensitive to the form of the dependence of magnification on wavelength. The large difference between quinine sulfate absorption maximum (350 nm) and the emission maximum (450 nm) makes quinine sulfate very sensitive to the wavelength dependence of the detection factor. Therefore measurements of the QY of fluorophores, which emit in the near UV, will require an accurate measurement of the dependence of the IS magnification on wavelength.

### 3.4 Estimates of Measurement Uncertainty

The uncertainties of QY given in Table 1 are the standard deviations (SD) of the results obtained from three independent measurements. These Type A uncertainties describe the repeatability of the entire sequence of four measurements of absorbance of buffer and analyte outside and inside the IS detector. In addition, it is useful to know the sensitivity of the QY values to uncertainties in each of the four measured absorbencies that enter into Eq. (7). The standard deviation (SD) of buffer absorbance measured outside and inside the IS detector was approximately 0.0002 absorbance units. This value was obtained from four independent consecutive measurements with 0.5 s integration time at each wavelength, and cuvette extraction and reinsertion after each measurement. The SD of the buffer absorbance measurement was consistent with the instrument noise. The SD values of absorbance of fluorescein solution with a nominal absorbance of 0.1 at 490 nm was 0.0003 outside and 0.0002 inside the IS respectively. Thus the SD of fluorescein absorbance measurements was less than 0.5 %. Assuming a change of 1 % in fluorescein absorbance measured outside the IS, Eq. (7) gives a change in QY of about 0.5 %. A change in absorbance of 1 % inside the IS gives a change of 1 % for the QY. The smaller sensitivity to *A*^out^ can be understood by examining Eq. (8) which shows that *A*^out^ affects both the numerator and the denominator in the same manner. Neglecting scattering (*r*_s_ = 0) in Eq. (7) changes QY by 1 %. Neglecting transmission (*t* = 1) in Eq. (7) changes QY by 4 %. Thus uncertainties in scattering and transmission estimates will have negligible effects on the value of QY of fluorescein. However the effects of scattering and transmission will be significant for fluorophores with small values of QY.

In addition to the four absorbance measurements, Eq. (7) also contains a factor describing the detection efficiency. In the case of fluorescein, the estimated detection efficiency factor in Eq. (7) increases the magnitude of the QY by about 16 %. The detection efficiency involves a product of the ratio of the PMT quantum efficiency and the ratio of IS magnification. In both cases the ratio is between values evaluated at absorbance and fluorescence wavelengths respectively. Thus relative quantum efficiencies and magnifications have a significant effect on the QY determined using Eq. (7). The uncertainty of the detection efficiency factor was not evaluated here but the close correspondence between the measured and accepted values of QY of fluorescein suggests that the estimate of the detection efficiency factor in Eq. (7) was reasonable.

## 4. Conclusion

This paper describes a method for determining the QY of fluorophores using a commercial spectrometer equipped with an integrating sphere (IS) detector. The measured values of the QY of fluorescein, rhodamine B, and erythrosin B are in agreement with established values. The value of QY obtained for quinine sulfate (maximum absorption at 350 nm) is very sensitive to the form of the wavelength dependence of the IS magnification. The wavelength dependence of the radiant cathode sensitivity of the PMT and magnification factors of the IS detector were estimated from generic data provided by the manufacturers. For a more accurate determination, the wavelength dependence has to be characterized for the specific instrument that is used for the measurements. This is especially true for wavelengths below 450 nm. In principle, the wavelength dependence of the magnification could be performed using molecules with large excitation bandwidth and QY = 1 [16]. The results obtained for QY are consistent with accepted values. An improved measurement model and characterization of the IS can lead to a robust and reliable method for determining QY in the UV-VIS. In practice it may be necessary to know only the wavelength of maximum fluorescence emission since a generic fluorescence emission spectrum that is shifted to the proper maximum wavelength can yield a sufficiently good estimate of the detection efficiency factor.

## Figures and Tables

**Table 1:** Quantum yield values obtained from application of Eq. (7), Eq. (7a), and Eq. (10)

Fluorophore	QY from Eq. (7)	Accepted value of Quantum yield
Myoglobin, PBS	−0.01	0.0
Fluorescein		
0.1 mol/L NaOH	0.90±0.02	0.925[14]
Rhodamine B, PBS	0.34±0.02	0.31
Erythrosin B, PBS	0.018±0.02	0.02[17]
Quinine sulfate		
0.1 mol/L H_2_SO_4_ solution	0.65	0.55[18]

PBS stands for phosphate buffer saline; the uncertainties were obtained from the SD of three measurements

**Table 2:** Relative cathode radiant sensitivity

Wavelength nm	Relative cathode radiant sensitivity
300	59.0
310	59.4
320	60.0
330	60.8
340	62.1
350	64.0
360	66.6
370	69.5
380	72.0
390	73.6
400	74.0
410	73.3
420	72.0
430	70.7
440	69.4
450	68.0
460	66.5
470	64.9
480	63.2
490	61.6
500	60.0
510	58.5
520	57.2
530	55.8
540	54.4
550	53.0
560	51.5
570	49.9
580	48.2
590	46.6
600	45.0
610	43.5
620	42.0
630	40.6
640	39.3
650	38.0
660	36.8
670	35.5
680	34.3
690	33.2
700	32.0

**Table 3:** Reflectance and magnification

Wavelength nm	Reflectance	Magnification
300	0.9565	15.3
310	0.9649	17.8
320	0.9714	20.2
330	0.9764	22.6
340	0.9801	24.8
350	0.9830	26.8
360	0.9852	28.5
370	0.9868	30.0
380	0.9881	31.2
390	0.9890	32.2
400	0.9898	33.0
410	0.9903	33.6
420	0.9907	34.1
430	0.9910	34.4
440	0.9913	34.7
450	0.9914	34.9
470	0.9917	35.2
480	0.9918	35.3
490	0.9918	35.4
500	0.9919	35.5

## Footnotes

1 Certain commercial equipment, instruments, and materials are identified in this paper to foster understanding. Such identification does not imply recommendation or endorsement by the National Institute of Standards and Technology.
